# Model Predictive Filtering MR Temperature Imaging for Laser‐Induced Interstitial Thermotherapy

**DOI:** 10.1002/mrm.70302

**Published:** 2026-02-15

**Authors:** Joshua Marchant, Robert J Bollo, Dennis L. Parker, Henrik Odéen

**Affiliations:** ^1^ Department of Radiology and Imaging Sciences University of Utah Salt Lake City Utah USA; ^2^ Harvard–MIT Division of Health Sciences and Technology Massachusetts Institute of Technology Cambridge Massachusetts USA; ^3^ Athinoula A. Martinos Center for Biomedical Imaging Massachusetts General Hospital Charlestown Massachusetts USA; ^4^ Department of Neurosurgery University of Utah Salt Lake City Utah USA

**Keywords:** accelerated acquisition, laser interstitial thermal therapy, magnetic resonance imaging, magnetic resonance temperature imaging, thermal modeling

## Abstract

**Purpose:**

To evaluate the use of the Model Predictive Filtering (MPF) method to improve temporal resolution of magnetic resonance temperature imaging (MRTI) for monitoring laser interstitial thermal therapy (LITT) ablations.

**Methods:**

Using a Green's function method for solving differential equations, a treatment‐specific power matrix *Q* was derived from a LITT heating and used in the Pennes bioheat equation (PBHE) to model a subsequent higher power heating and supplement subsampled *k*‐space data. This MPF method was evaluated using both 3D segmented EPI data and a tissue mimicking phantom and clinical LITT treatment data after retrospective subsampling. Reconstruction accuracy was assessed via thermal dose and analysis of the hottest voxel and region‐of‐voxels over time.

**Results:**

In the phantom data, temporal resolution equivalent to a 12‐slice acquisition was produced with larger fields‐of‐view (24 and 36 slices, *R* = 2 and 3) with good hottest voxel‐over‐time accuracy and 240 CEM_43_ volume agreement (Dice similarity coefficient, DSC ≥ 0.7). In the in vivo data, MPF reconstruction showed excellent 240 CEM_43_ volume agreement for both orthogonal slices (DSC > 0.9 for *R* = 2 and 3). The sagittal and coronal slices showed excellent hottest voxel accuracy for subsampling of *R* ≤ 3, with an RMSE ≤ 1°C. Hottest voxel RMSE remained within 1°C–3°C up to a subsampling factor of 5.

**Conclusion:**

The MPF algorithm allowed for large field‐of‐view (FOV) volumetric temperature imaging without decreasing temporal resolution in phantom heatings. Bi‐planar clinical treatment data reconstruction showed good accuracy for the application of MPF to in vivo data.

## 1 Introduction

Laser interstitial thermotherapy (LITT) is a minimally invasive, surgical modality used to treat a variety of indications, including brain tumors, spinal metastases, and epilepsy [1, 2, 3]. LITT utilizes a surgically inserted laser‐emitting catheter at high powers (12–15 W) [4], designed to ablate target tissues in clinically feasible times. Modern LITT procedures are often monitored using magnetic resonance temperature imaging (MRTI). To satisfy spatio‐temporal MRTI acquisition constraints required for real‐time tissue temperature monitoring, clinicians are currently forced to rely on multi‐slice two‐dimensional images during the procedures. Without an estimation of the volumetric temperature distribution, clinicians can be severely limited in their ability to assess the full status of the ablative procedure.

Multiple approaches have been investigated to accelerate MRTI acquisitions [5]. An accelerated acquisition can be used to image an enlarged field‐of‐view (FOV) or to increase the temporal resolution. Faster acquisitions can be achieved using faster pulse sequences, such as echo planar imaging (EPI), where multiple lines of *k*‐space are acquired after each RF pulse [6]. For LITT applications, this has been investigated for both multi‐slice 2D and volumetric 3D imaging [7]. An alternative approach to accelerate imaging is to only acquire portions of *k*‐space and use dedicated reconstruction algorithms to reconstruct artifact free images. One such approach is parallel imaging, where the spatial sensitivity from multiple RF receive coils are used to suppress aliasing artifacts. This approach can be performed both in image space and in *k*‐space and has been extensively investigated and used in LITT applications [8, 9, 10]. LITT lends itself well to parallel imaging approaches as standard head and flex coils can typically be fitted around the anatomy of interest even when the LITT fiber is present. Two other groups of reconstruction approaches for subsampled MRTI data are model‐based and compressed sensing‐based approaches [8, 11, 12, 13].

In this work, we propose the use of a model‐based approach, called model predictive filtering (MPF), to reconstruct subsampled *k*‐space data. The MPF method, described in detail in a previous work [14], combines acquisition of subsampled data with a thermal model which forward‐predicts the heating process. The predicted heating can be turned into predicted *k*‐space samples and used to fill in missing data in *k*‐space. In high‐intensity focused ultrasound (HIFU) applications, the MPF method has been shown to successfully produce large FOV temperature maps with sampling reduction factors as high as *R* = 7.1 for 3D applications (RMSE ≤ 0.61°C). In the HIFU case, the thermal model utilized was based on Pennes Bioheat Equation (PBHE) [15, 16, 17, 18]. Here, the deposited energy, *Q*, has been derived using pixel‐wise linear and analytical fitting to the temperature curve over time [19], acoustic simulations [20], or by solving the PBHE for *Q* using Green's function for differential equations [21]. The MPF approach was shown to be relatively robust against model inaccuracies: 50% errors in thermal conductivity λ and deposited power *Q* resulted in a maximum temperature error of 3°C. Improved modeling methods achieved comparably low error for increased reduction factors (RMSE < 0.7°C for *R* = 12) [22].

To apply the MPF method, the deposited power density *Q* must be estimated as an input to the PBHE. A limited number of strategies have been proposed for modeling the deposited power in LITT procedures. Fuentes et al. proposed a 2D finite element model (FEM) based on two optical photon source approximations [9]. Farenholtz et al. compared a 2D FEM‐based model (similar to the model provided by Fuentes et al. but with optimized parameter selection) with one example of a steady‐state analytical solution to the PBHE that provides an approximation of heat propagation from a single point source [23]. They found that both models provided reasonable results after model optimization but could benefit from further investigation and refinement. A follow‐up study investigated the use of a steady‐state analytical solution in 2D heterogeneous tissues [24].

Despite the promising results of such strategies in 2D, variations in laser and catheter design can be difficult and computationally expensive in 3D to model across laser system manufacturers. For example, the Visualase system (Medtronic, Louisville, CO, USA) has a laser diffusing tip with a diameter of 1.65 mm and tip lengths of 3 and 10 mm, 15 CW non‐pulsed output, and saline cooling, while the NeuroBlate system (Monteris, Winnipeg, Canada) offers both diffusing (FullFire system) and directional (SideFire system) laser emitters with diameters of 2.2 and 3.3 mm, ≤ 12 W pulsed output, and CO_2_ cooling [4]. In addition to the variations in laser fibers, patient‐specific probe positioning, including proximity to blood vessels which can act as significant heat sinks [21, 25], also complicates accuracy of FEM‐only based approaches. To alleviate these potential challenges, we propose the use of the Green's function‐based solution to the PBHE to model a 3D heat deposition matrix, *Q*, using MRTI data from a low‐power test heating as input. Because the *Q* matrix is derived directly from a low‐power test heating, this strategy is inherently system and patient specific. The *Q* matrix can be used again in the PBHE to forward model the temporally and spatially varying temperature rise at the target. We performed experiments in tissue mimicking phantoms using subsampled 3D gradient recalled echo (GRE) segmented EPI data, and in vivo using retrospectively subsampled data from multi‐slice 2D GRE acquisition from a standard of care LITT treatment.

A physician performing a LITT procedure typically uses the information from both a thermal dose map and temperature over time curves at various points of interest to monitor the progress of the procedure. Therefore, the data was analyzed in terms of thermal dose maps and temperature over time curves for the hottest voxel (or 3 × 3 region of voxels in vivo). The dose maps were defined as regions of voxels where 240 cumulative equivalent minutes at 43°C (240 CEM_43_) were reached [26, 27]. While other thermal dose models exist, such as a temperature threshold [28] or the Arrhenius rate process [29], Yung et al. found that all of the aforementioned models can operate reliably as a thermal dose measure for treatment evaluation [30]. Similar to Yung et al. a comparison of 240 CEM43 regions was performed using the Dice similarity coefficient (DSC). In addition to analyzing the point of heating, three additional points (two safety points and one drift monitoring point) were identified by the performing physician (RB) and included in the proof‐of‐concept clinical study.

## 2 Methods

### 2.1 Solving the Bioheat Transfer Equation Using Green's Function Heat Kernel

The PBHE is defined as

(1) $$\rho c\frac{\partial T}{\partial t}=\lambda \nabla^{2}T-\mathrm{wc}\left(T-T_{\mathrm{ar}}\right)+Q$$

where T is the temperature distribution (°C) in tissue at the selected input time point $T\left(x,y,z,t_{\text{in}}\right)$, $T_{\mathrm{ar}}$ is the arterial blood temperature (°C), ρ is the tissue density (kg/m^3^), c is the specific heat of the tissue (J/kg/°C), λ is the tissue thermal conductivity (W/m/°C), w is the Pennes blood perfusion constant (kg/m^3^/s), and *Q* is the heat deposition per unit volume (W/m^3^) [14, 15, 16, 17, 18]. The PBHE can be solved for *Q* using a Green's function for differential equations using the method presented by Freeman et al. and others [21, 31]. Applying this strategy (described in more detail in Freeman et al. [21]), the specific absorption rate (SAR, in W/kg) can be estimated from MRTI data by:

(2) $$\mathrm{SAR}=c{ℱ}^{-1}\left(\frac{\hat{T}-\hat{f}\hat{K}-\left(w/\rho \right)ℱ\left(T_{\mathrm{ar}}\times K\right)}{\int_{0}^{t}\hat{K}\left(k_{x},k_{y},k_{z},\tau \right)d\tau \;}\right)$$

As seen in Equation (2), SAR is calculated using the Fourier transforms of the following variables: T (denoting $T\left(x,y,z,t_{\text{in}}\right)$ as above), f(x,y,z) which denotes the initial temperature distribution $T_{0}\left(x,y,z,t=0\right)$, and K as the heat kernel, defined as

(3) $$K={\left(\frac{1}{2\sqrt{\pi \kappa t}}\right)}^{3}\exp\left(-\frac{x^{2}+y^{2}+z^{2}}{4\kappa t}\right)\exp\left(-\frac{w}{p}t\right)$$

where κ is the thermal diffusivity ($m^{2}/s$; κ=k/ρλ). When the perfusion w is assumed to be zero, Equation (2) simplifies to

(4) $$\mathrm{SAR}=4\pi^{2}\kappa c{ℱ}^{-1}\left(\frac{\hat{T}-\hat{f}\exp\left(-4\;\pi^{2}\kappa t{\left|k\right|}^{2}\right)}{1-\exp\left(-4\pi^{2}\kappa t{\left|k\right|}^{2}\right)\;}{\left|k\right|}^{2}\right)$$

If the tissue density is known or can be estimated, *Q* can be derived numerically from a discrete time‐varying spatial temperature distribution after time t,considering that

(5) $$\mathrm{SAR}=\frac{Q}{\rho }$$

The above derivation can be applied equivalently in two dimensions. Note that for all experiments, the initial temperature f(x,y,z) was considered to be a constant scalar value, such that temperature changes were found *relative* to this constant baseline. This baseline value is arbitrary and could flexibly be set to, e.g., 37°C, 20°C, or 0°C depending on if relative or absolute temperature changes are of interest.

### 2.2 Applying Model Predictive Filtering

The MPF algorithm is described in detail in previous work [14], but the main steps and how they relate to the present study are described briefly here for the benefit of the reader.

#### 2.2.1 Estimation of *Q*

**FIGURE 1:**
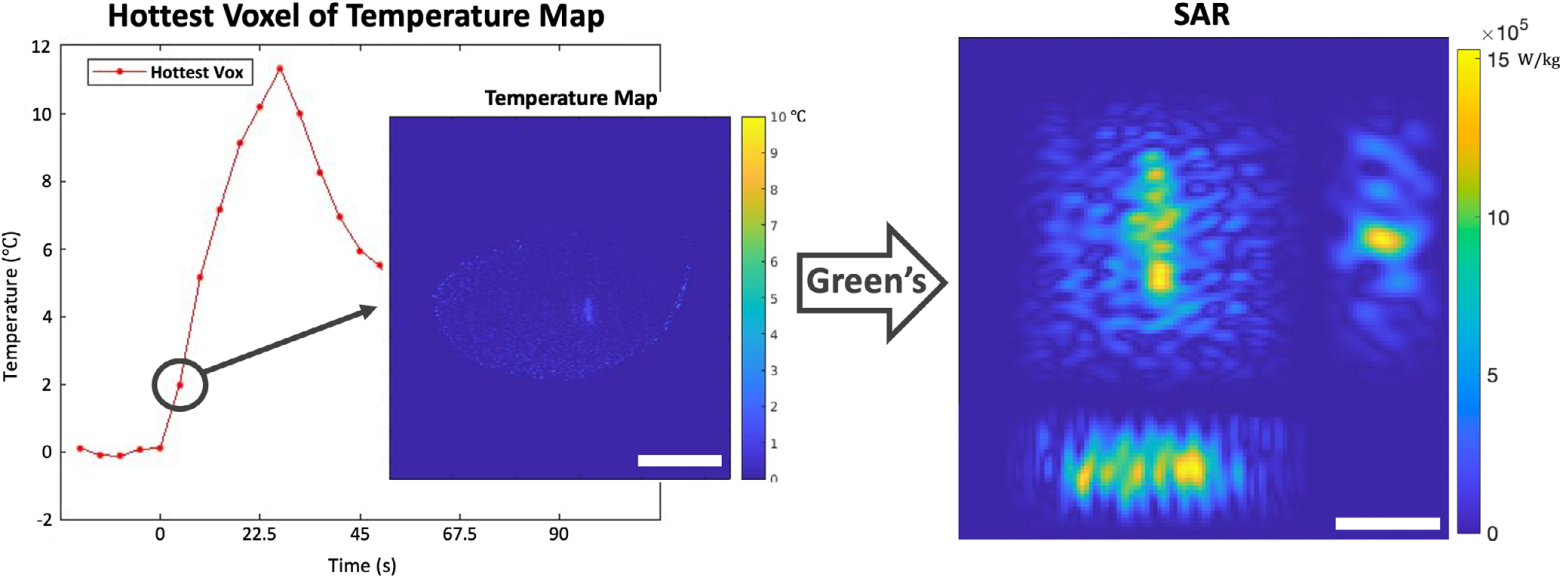
Diagram showing the process to obtain *Q* matrix from a low‐power heating. Here, the hottest voxel‐over‐time curve (red) is shown for a low power phantom heating. The 3D temperature distribution shown by the black circle and arrow, and temperature image (left), the Green's function is deconvolved, and a 3D SAR matrix is obtained (right; three orthogonal views of the pattern are shown). *Q* can be obtained using Equation (5). Scale bars are 50 mm (temperatures) and 10 mm (SAR).

The first step involves the estimation of *Q*, which is needed to calculate heat transfer via the PBHE. In the phantom study, a 3D *Q* matrix was derived using a low power heating, similar to previous work for HIFU applications [14, 20, 22]. This low power heating was monitored with a fully sampled (i.e., no *k*‐space subsampling) small FOV (12 slices in the 3D volume, Table 1) acquisition, to provide a temperature map with high temporal resolution. This temperature map was used as the input to the Green's function, described in Equations ((1), (2), (3), (4), (5)) above and in Figure 1. Freeman et al. note that using the first heating dynamic when deriving *Q* produces the lowest error in the estimation [21], so this principle was employed in the present work [18]. Using the first time point/low power for this “calibration” heating further ensures safety by delivering minimal heating. For the phantom data, the 3D *Q* matrix was derived from the first heating time point which had a maximum heating temperature of approximately 2°C. For the in vivo study, data was obtained from a clinical treatment after informed consent and corresponding Institutional Review Board (IRB) approval. No low power heating was available and 2D *Q* matrices were derived from 2D MRTI data. In this case, *Q* was derived from a fully sampled separate heating run in the same patient, previous to the heating run used to evaluate the MPF algorithm. Here the second heating dynamic, with a maximum heating temperature change < 10°C, was utilized for both slices to ensure the temperature measurement was distinguishable from the noise levels of in the in vivo data, which are reported below.

**TABLE 1:** Acquisition details for the phantom and clinical data.

	Description	FOV (mm)	Matrix	Resolution (mm)	Acq. Time (s)	Power (W)	Reduction factor
Three phantom experiments	Low‐Power	231 × 217 × 30	256 × 120 × 12	0.9 × 1.8 × 2.5	4.5	4	—
	“Truth” 1	231 × 217 × 30	256 × 120 × 12	0.9 × 1.8 × 2.5	4.5	8	—
	Large FOV 1	231 × 217 × 60	256 × 120 × 24	0.9 × 1.8 × 2.5	9	8	2
	Large FOV 2	231 × 217 × 80	256 × 120 × 32	0.9 × 1.8 × 2.5	13.5	8	3
	“Truth” 2	231 × 217 × 30	256 × 120 × 12	0.9 × 1.8 × 2.5	4.5	8	—
In vivo data	2 orthogonal slices (sagittal and coronal)	240 × 240	256 × 256	0.94 × 0.94 × 3	6.4	4.5–12	2 or 3

*Note*: The set of 5 MRTI runs for the phantom listed in the Table was repeated three times, for a total of 15 scans. Note that values shown are previous to zero‐filled interpolation (to 0.9 mm isotropic resolution), described further in the methods.

Due to inaccuracies in the initially determined *Q*, temperature estimations from the PBHE can be over‐ or under‐estimated [21]. To alleviate this problem, the estimated *Q* was used in the PBHE to simulate a temperature map which was then scaled based on the hottest voxel such that the maximum simulated temperature was equal to that of the test heating from which *Q* was found. The resulting scaled *Q* was cropped to a region of interest (ROI) around the laser probe to avoid the edge of the phantom (for ex vivo data) and the skull and air interfaces (for in vivo data). This can be done as PBHE modeling is only needed in the region where the heating is occurring. A 3D median filter was applied to *Q* prior to scaling to reduce noise.

#### 2.2.2 Calculation of Phase Change at Time Step *n* + 1

Once *Q* is estimated, it can be utilized in the PBHE along with the laser power input (in W) and literature values for all thermal properties for the phantom and brain, respectively, as follows: gellan gum/brain density: 1030/1046 kg/m^3^, specific heat: 4186/3630 J/kg/°C, thermal conductivity: 0.56/0.51 W/m/°C, and no perfusion. The perfusion was set to 0 as any perfusion effects on heating were implicitly included in the calculation of *Q* as the MRTI used as input for *Q* calculation reflected temperature changes based on both laser heating and physiological contributors. These parameters were used to produce a fully simulated 2D/3D temperature map prediction for the next time point, *n* + 1. This temperature map was then converted to an estimated phase shift map using the proton resonance frequency (PRF) shift equation [32, 33]:

(6) $$\Phi_{n+1}=\Phi_{n}+\gamma B_{0}\alpha \mathrm{TE}\left(T_{n+1}-T_{n}\right)$$

where $\Phi_{n}$ is the phase at time frame *n* (radians), γ is the gyromagnetic ratio (MHz/Tesla), $B_{0}$ is the magnetic field strength (Tesla), α is the PRF coefficient (assumed to be −0.01 ppm/°C in this work) and TE is the echo time of the acquisition (milliseconds). This simulated phase map at time *n* + 1 was combined with the acquired magnitude image at time *n* and transformed to *k*‐space using a Fast Fourier Transform (FFT). The fully sampled, partly model‐generated, *k‐*space was used to fill in the missing lines of the acquired, subsampled, *k‐*space at time *n* + 1 to create a fully sampled, “model‐supplemented” *k‐*space at time *n* + 1, which was transformed back to image space using an Inverse Fast Fourier Transform (IFFT). The phase of this complex image was then converted to an updated temperature map at time *n* + 1, again using the PRF equation. This process was repeated for all *N* time points of the full experiment.

During LITT treatments, coolant, in this case room temperature saline, is flown in the catheter around the laser probe to prevent too high temperatures from occurring close to the probe. This could be modeled in the current PBHE implementation; however, we found that the most accurate temperature models were achieved by simply using the derived *Q* without additional modeling of the cooling. This may be due to the difficulty in accurately modeling an active cooling system with limited MRTI data resolution and is addressed further in the discussion.

### 2.3 Computational Environment

All data reconstruction, modeling, and analysis was performed in MATLAB (Mathworks, Natick, MA, USA). A lab‐hosted compute box was used (CentOS Linux, 2.10 GHz Dual 16 core Intel Xeon CPU E5‐2620 v4, 512 Gb RAM) for modeling and image reconstruction of the 3D phantom data. The reconstruction time and model simulation time using MPF for the entire 25 time‐point volumetric data was 26.1 s, averaging 1.0 s per time point. For the 2D in vivo data, a standard laptop (MacBook Pro, Apple, Cupertino, CA, 1.4 GHz Intel Core i5, 8 GB RAM) was used. The reconstruction time and model simulation time using MPF for the entire 25‐time point 2D set was 1.5 s, averaging 3.75 ms per time point. Note that in both cases, unoptimized code in a high‐level language (MATLAB) was utilized for these proof‐of‐concept tests. Compiled, optimized code would be expected to produce even faster results.

### 2.4 Phantom Study

For the phantom study, three identical sets of five MRTI‐monitored LITT heatings (Visualase, Medtronic, Louisville, CO, USA), that is, a total of 15 heatings, were performed in an in‐house made gellan gum brain phantom [7]. The treatment protocol for one such set is described in Table 1. The tissue‐mimicking gellan gum (Fisher Scientific, Pittsburgh PA; 10 g Gellan gum powder per 1000 mL deionized water) was made in a hollow plastic skull (Model A20, 3B Scientific, Tucker, GA) to approximate the geometry and size of a human adult cranium. All MRTI acquisitions were performed using a GRE segmented EPI sequence, with FOV 231 × 217 mm (256 × 120 voxels) in plane, 12, 24, or 36 slices, 0.9 × 1.8 × 2.5 mm resolution, 21/12 ms TR/TE, 850 Hz/px bandwidth, 10‐degree flip angle, echo train length of 5, 6/8 partial Fourier in kz, and 4.5 s acquisition time for 12 slices, 9 s for 24 slices and 13.5 s for 36 slices on a 3 T scanner (Prisma^FIT^, Siemens Healthineers, Erlangen, Germany). In all cases, heating lasted for a total of 27 s (3 dynamics for 24 slices, 2 dynamics for 36 slices before subsampling). A 4‐channel flex coil (Siemens Healthineers) was used, and multi‐coil data was optimally combined using Roemer's equation (via Equation 24 from Roemer et al. [34]; see also work from Parker et al. [35] for a full explanation). All images were zero‐filled interpolated to 0.9 × 0.9 × 0.9 mm resolution.

As noted previously, a separate low‐power 4 W heating was used to estimate *Q* for each of the three repeated sets. The 4 W heatings were acquired over the smaller FOV (231 × 217 × 12 voxels) to match the scan time of the “truth” heatings. It can be noted that while this 4 W heating resulted in a temperature rise of ˜11°C, the first heating time point, with a temperature rise of only ˜2°C, was used to find *Q*. To account for possible hysteresis in temperature rise over consecutive heatings, a “truth” heating was acquired before and after each of the two Large FOV MPF heatings and averaged within each set. After the low‐power heating and the first “truth”, the two larger FOV heatings (with 24 and 36 slices) were acquired at a lower temporal resolution, Table 1. These two heatings were retrospectively subsampled with *R* = 2 and *R* = 3, respectively, and reconstructed with the MPF approach as described above to achieve the same temporal resolution as the 12‐slice “truth” acquisitions. Retrospective subsampling of the phantom data was performed according to the kz‐inside‐ky sampling order (i.e., one echo train was acquired for all kz slice encoding lines, before proceeding to acquire the next echo train for all kz slice encoding lines, and so on) of the segmented EPI sequence.

Measurement precision was determined by defining a background ROI in the phantom where no heating occurred, and calculating the (averaged) voxel‐wise standard deviation through time. Accuracy was determined by measuring the voxel‐wise RMSE in the same ROI with respect to 0°C. Phantom temperature maps were found to have a precision and accuracy of 0.2°C.

### 2.5 In Vivo Application

MRTI data was acquired from a pediatric case (10‐year‐old male) being treated for epilepsy after IRB‐approved informed consent. The laser applicator was stereotactically inserted and laser ablation of a left frontal region of focal cortical dysplasia was performed. As the clinical system (Visualase) does not support 3D MRTI, the clinically used 2 orthogonal 2D slices placed along the laser fiber were acquired from the LITT treatment. This in vivo dataset served to illustrate the efficacy of using the MPF algorithm in a clinical setting.

The data were acquired using a standard 2D spoiled gradient echo sequence (FOV 240 × 240 mm, matrix size 256 × 256 voxels, 0.94 × 0.94 × 3 mm resolution, 25/15 ms TR/TE, 78 Hz/px bandwidth, 30‐degree flip angle, and 6.4 s acquisition time for two orthogonal slices) on a 1.5 T scanner (SIGNA Artist, GE Medical Systems, Chicago, Illinois). A 4‐channel flex RF receive coil (GE Medical Systems) was used. Similar to the phantom data, the in vivo data were reconstructed using an in‐house developed MRTI reconstruction pipeline. No zero‐filling was performed.

The sagittal and coronal slices were reconstructed and analyzed separately. In contrast to the phantom data, the power level applied during the treatment was not constant. Based on the clinical treatment record, a discretized vector of power values was used in the PBHE. During the heating run used in the MPF, a power of 4.5 W was applied for approximately 19.2 s, 11.25 W for 38.4 s, and 12 W for 32 s, for a total heating time of approximately 90 s. Subsampling for the in vivo patient data was performed retrospectively using a pseudo‐random, pseudo‐Gaussian subsampling pattern. In short, for each time point, the “preserved” *k*‐space lines not eliminated during the subsampling were chosen using a normal probability distribution function (via the “normpdf” function in MATLAB), where the center of *k*‐space was used as the mean of the PDF. These *k*‐space lines were sampled until reaching the appropriate number of fully sampled lines for each subsampling factor, which ranged from *R* = 2 to *R* = 5. We consider the sampling to be “pseudo‐Gaussian” because the middle 20 lines (out of 256 total phase encoding lines) were *always* preserved, ensuring the center of *k*‐space was fully sampled. Several trials indicated that a pseudo‐random selection of subsampled *k*‐space lines was ideal to ensure that the model was supplementing different *k*‐space regions for *each* time point, such that any possible model bias produced by a given *k*‐space supplementation was incoherent over time. Figure S3 provides a visualization of one time point for subsampling with *R* = 2 and *R* = 5.

Measurement precision and accuracy were determined in the same manner as the phantom data. The clinically obtained temperature maps were found to have a precision and accuracy of 0.7°C and 0.8°C (sagittal slice) and 0.5°C and 0.6°C (coronal slice) respectively.

### 2.6 Analysis

In both the phantom and in vivo study, logical ROI were defined where 240 cumulative minutes at 43°C (CEM_43_) were reached in both the MPF and “truth” (for the phantom study) or fully sampled data (for in vivo scan) temperature maps. During the calculation of the CEM_43_, both the patient and the phantom were assumed to have an initial temperature of 37°C (the phantom was really at room temperature of ˜21°C). The overlap between the MPF and “truth” CEM_43_ regions of interest was compared using the DSC. The DSC has previously been employed to quantify the agreement between complex segmented image regions and regions of thermal damage [30, 36, 37]. A DSC value greater than 0.7 has previously been considered to indicate good spatial agreement between the two areas or volumes [30]. Given the different number of slices, only the overlapping slices between the MPF data and “truth” could be compared for the phantom study.

Additionally, the behavior of the hottest voxel (or regions of voxels) was plotted for visual comparison of the MPF and “truth” data. For the phantom data, only the single hottest voxel in each data matrix was considered as the SNR was higher. For the in vivo data, a 3 × 3 ROI of voxels centered about the hottest voxel was used for comparison.

When performing a LITT procedure, the physician commonly observes the temperature over time at nearby sensitive structures using “safety points” to ensure that dangerous levels of heating are not observed during the treatment. Additionally, a drift monitoring point is often selected in the contralateral hemisphere. Therefore, a 3 × 3 ROI analysis over time was also performed at the following regions, selected by the performing physician (RB): the corpus callosum (sagittal slice), the pericallosal artery (coronal slice), and a drift check in the contralateral hemisphere (coronal slice).

All MPF and “truth” temperature‐over‐time data were compared using a two‐sample *t*‐test assuming independence.

## 3 Results

### 3.1 Phantom Study

**FIGURE 2:**
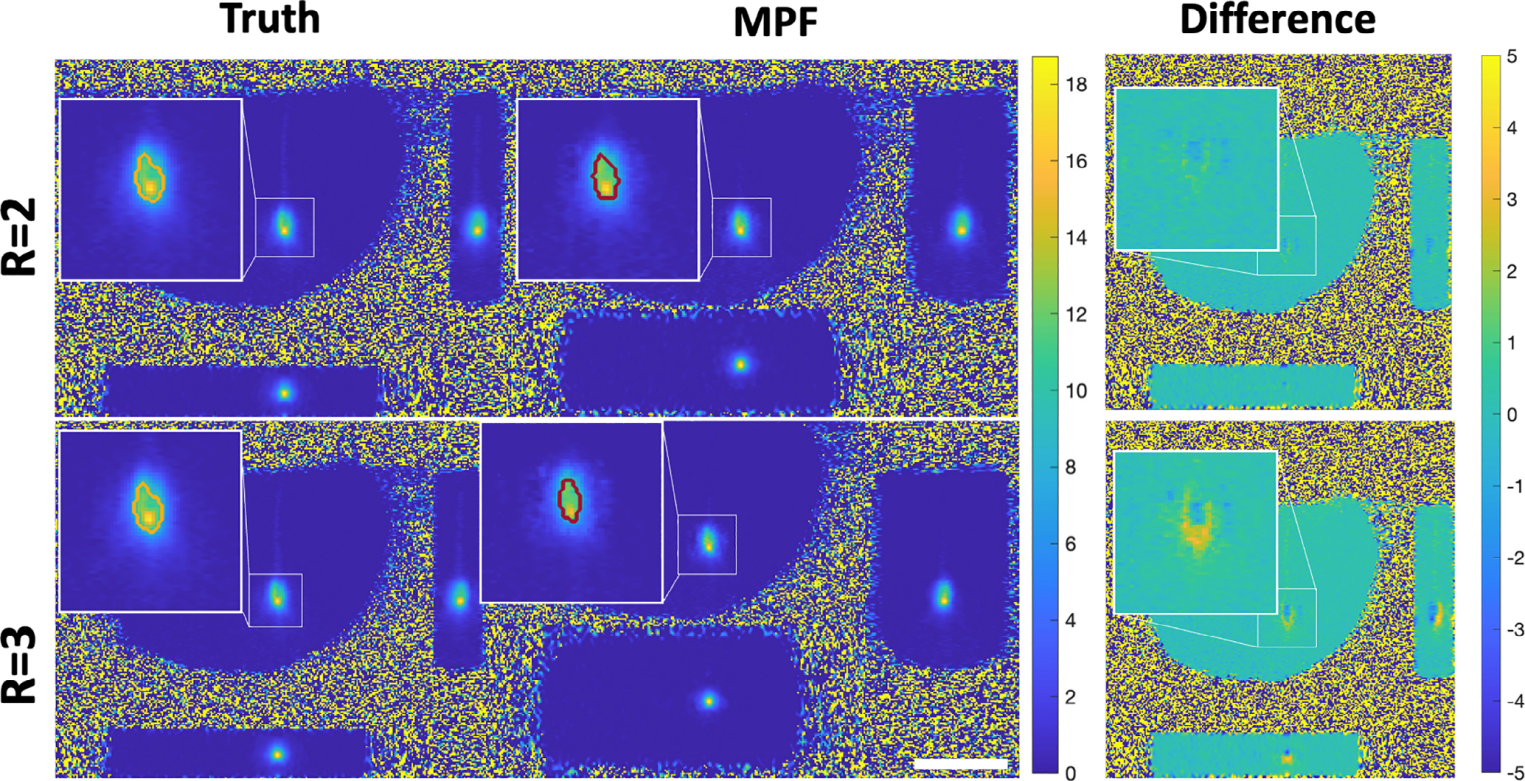
The second set of phantom “truth” and MPF reconstructed temperature maps (3D) at the hottest time point with overlayed 240 CEM_43_ contours. Rightmost images show the difference maps, where Difference = Truth−MPF. Three orthogonal views are shown for each trial (center: XY, right: XZ, bottom: YZ). Scale bar is 50 mm.

**FIGURE 3:**
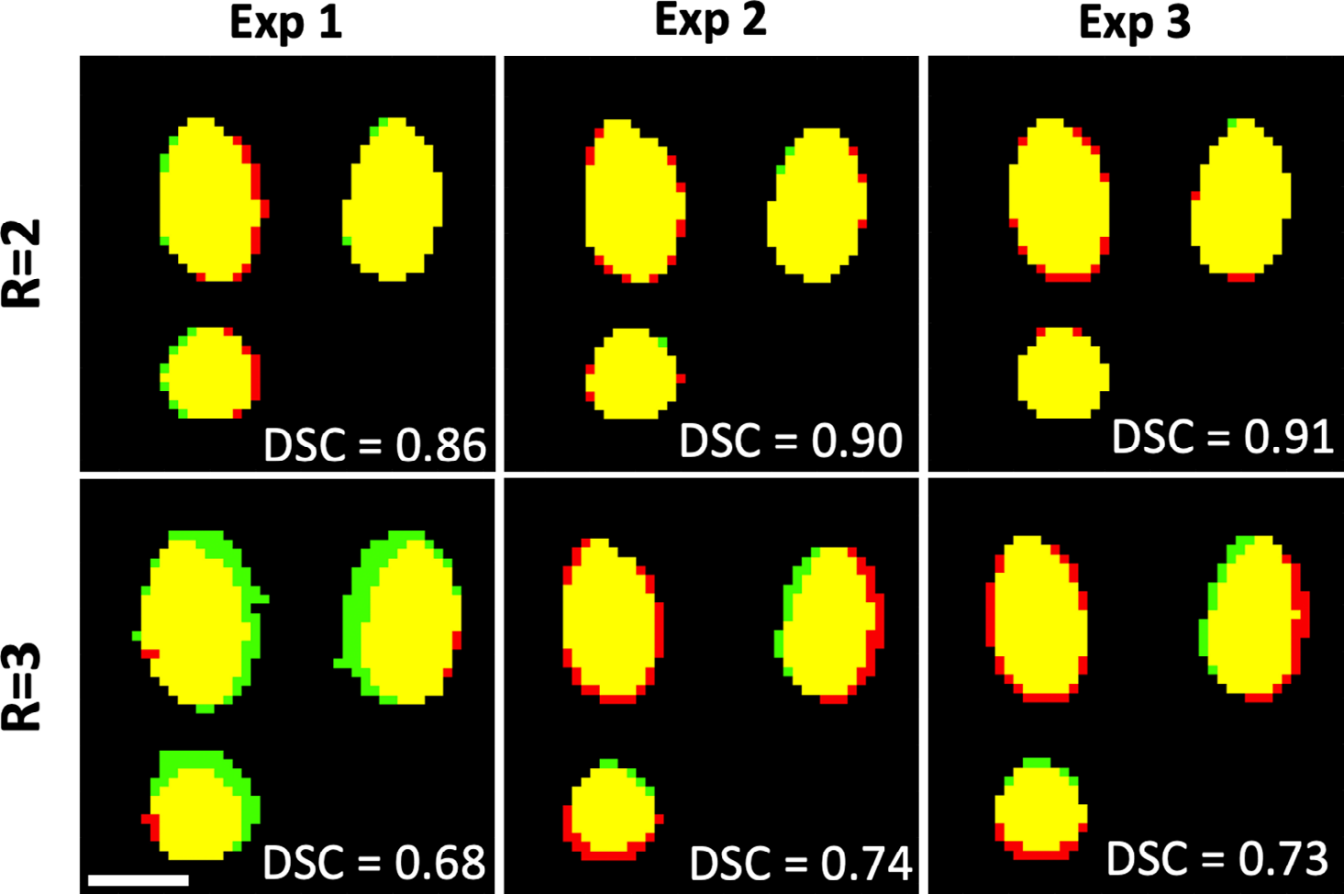
Comparison of the 240 CEM_43_ volume for all six phantom data sets, with DSC scores listed. Yellow indicates matching regions, while red shows “truth” only and green shows MPF only. Three orthogonal views are shown for each volume (top left: XY, top right: XZ, bottom left: YZ). Scale bar is 10 mm.

Figure 2 shows three orthogonal views of the volumetric temperature data for Experiment 2 at the hottest slice and dynamic for both subsampling factors. Images from Experiment 1 and 3 can be found in the Figures S1 and S2. The overlayed contours indicate the outline of the 240 CEM_43_ region at the slice and time point of maximum heating. In all six data sets, 240 CEM_43_ regions between the MPF and “truth” showed good agreement. For the *R* = 2 data sets, the DSC values were 0.86, 0.90, and 0.91; for the *R* = 3 data, the DSC values decreased to 0.68, 0.74, and 0.73, Figure 3, that is, 5/6 runs met the performance of having a DSC above 0.7. High spatial agreement can be seen in the orthogonal views in Figure 3 for *R* = 2. For *R* = 3, experiments 2 and 3 show good agreement while experiment 1 has greater discrepancies at the edges. As seen in the difference images (See Figure 2 and Figures S2 and S3), the MPF temperatures at the hottest time point were overestimated near the center of the heating and slightly underestimated near the tip and periphery of the laser. Such discrepancies were more pronounced in the *R* = 3 data.

**FIGURE 4:**
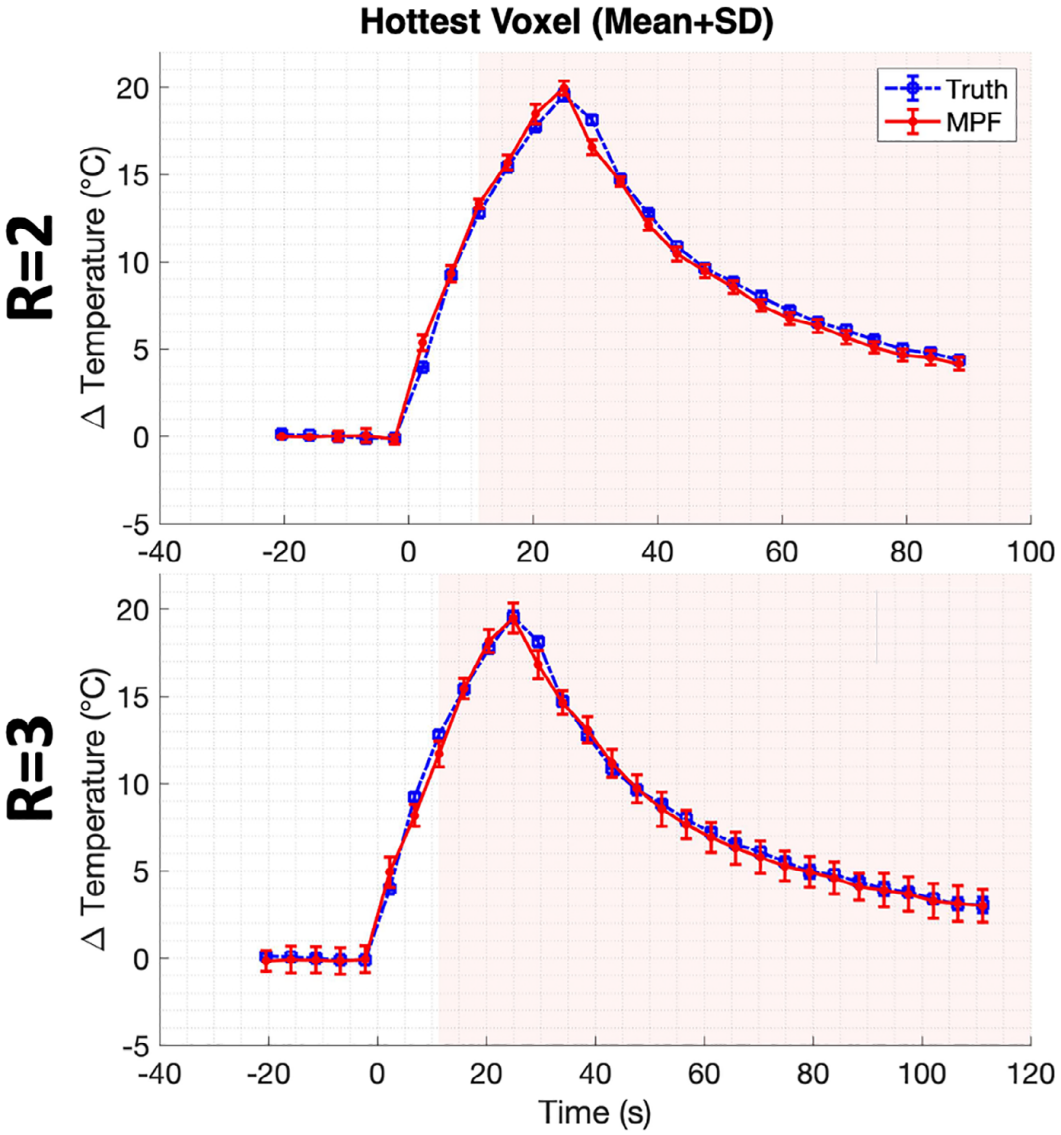
Temperature curves for the hottest voxel‐over‐time, averaged over all three experiments per subsampling factor. Both curves show close agreement with increased error near the laser‐on and laser‐off regions. *R* = 3 showed an increase in SD between experiments.

The hottest voxel in the MPF and “truth” data was averaged over all three experiments for each subsampling factor *R* (± standard deviation, SD) and plotted over time in Figure 4. Both the *R* = 2 and *R* = 3 averaged data sets showed good agreement, with a slight increase in SD using *R* = 3. For both subsampling factors, the greatest error in the averaged MPF data occurred within the first three time points of the laser turning on (dynamic one for *R* = 2, error = 1.4°C; dynamic three for *R* = 3, error = 1.1°C) and the first time point after being turned off (dynamic seven for both, error = 1.7°C, *R* = 2 and 1.3°C, *R* = 3). All other time points during heating resulted in an error < 1°C. The RMSE values at the hottest voxel over time for the *R* = 2 data sets were 0.8°C, 0.5°C, and 0.6°C; and for *R* = 3, 1.0°C, 0.7°C, and 0.8°C, for the three Experiments, respectively. In both cases, RMSE values were slightly higher than the measurement accuracy, suggesting a small increase in error ($\leq 0.8^{{}^{\circ}}C$) due to the MPF implementation. No significant difference (*p* > 0.05) was found between the MPF and “truth” hottest voxels over time in all six data sets using a two‐sample *t*‐test.

### 3.2 In Vivo Data

**FIGURE 5:**
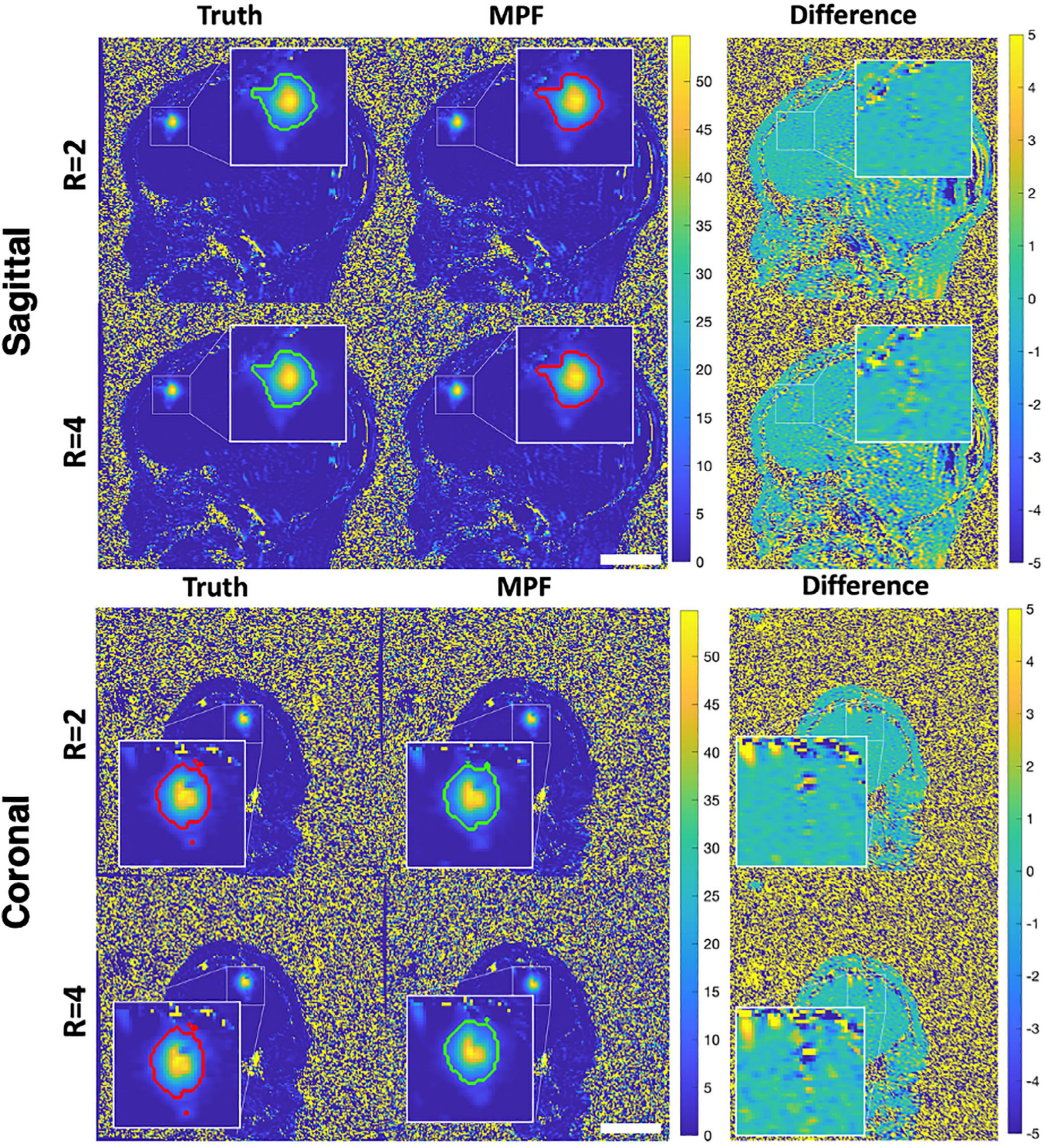
The sagittal and coronal slices of in vivo “truth” and MPF reconstructed temperature maps (2D) with overlayed 240 CEM_43_ contours are shown. Rightmost images show the difference maps, where Difference = Truth−MPF. Scale bars are 50 mm.

**FIGURE 6:**
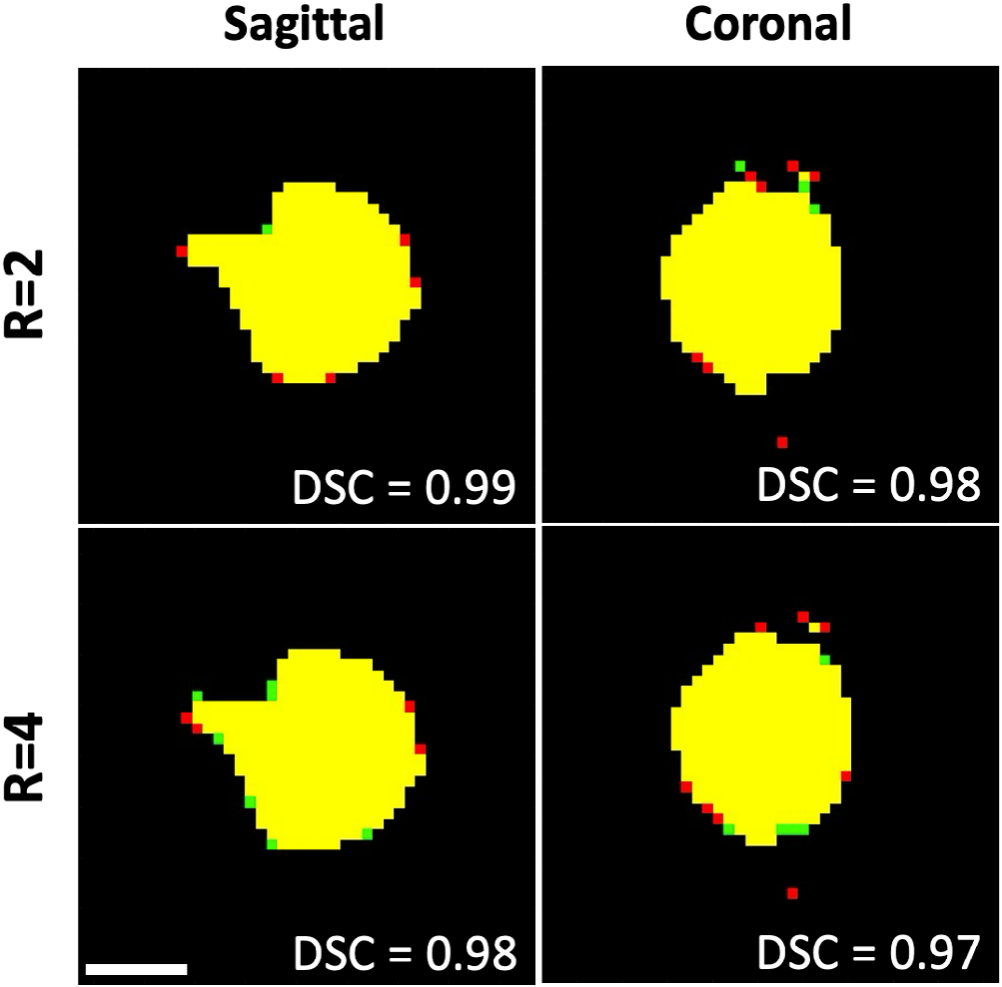
Comparison of the 240 CEM_43_ area for both bi‐planar slices at *R* = 2 and *R* = 4, with DSC scores listed. Again, yellow indicates matching regions, while red shows “truth” only and green shows MPF only. Scale bar is 10 mm.

Figure 5 shows cropped regions of the temperature images for both *R* = 2 and *R* = 4 for the sagittal and coronal slices. Again, the 240 CEM_43_ regions are illustrated by the overlayed contours. As observable in the difference images to the right, a pattern of alternating positive and negative errors (not seen in the phantom images) occurred for both orientations. The comparison of the MPF and “truth” 240 CEM_43_ areas is shown in Figure 6. The DSC values indicate excellent agreement between the 240 CEM_43_ volumes for both *R* = 2 and *R* = 4 subsampling factors at both orientations, with DSC of 0.97–0.99 for all image sets.

**FIGURE 7:**
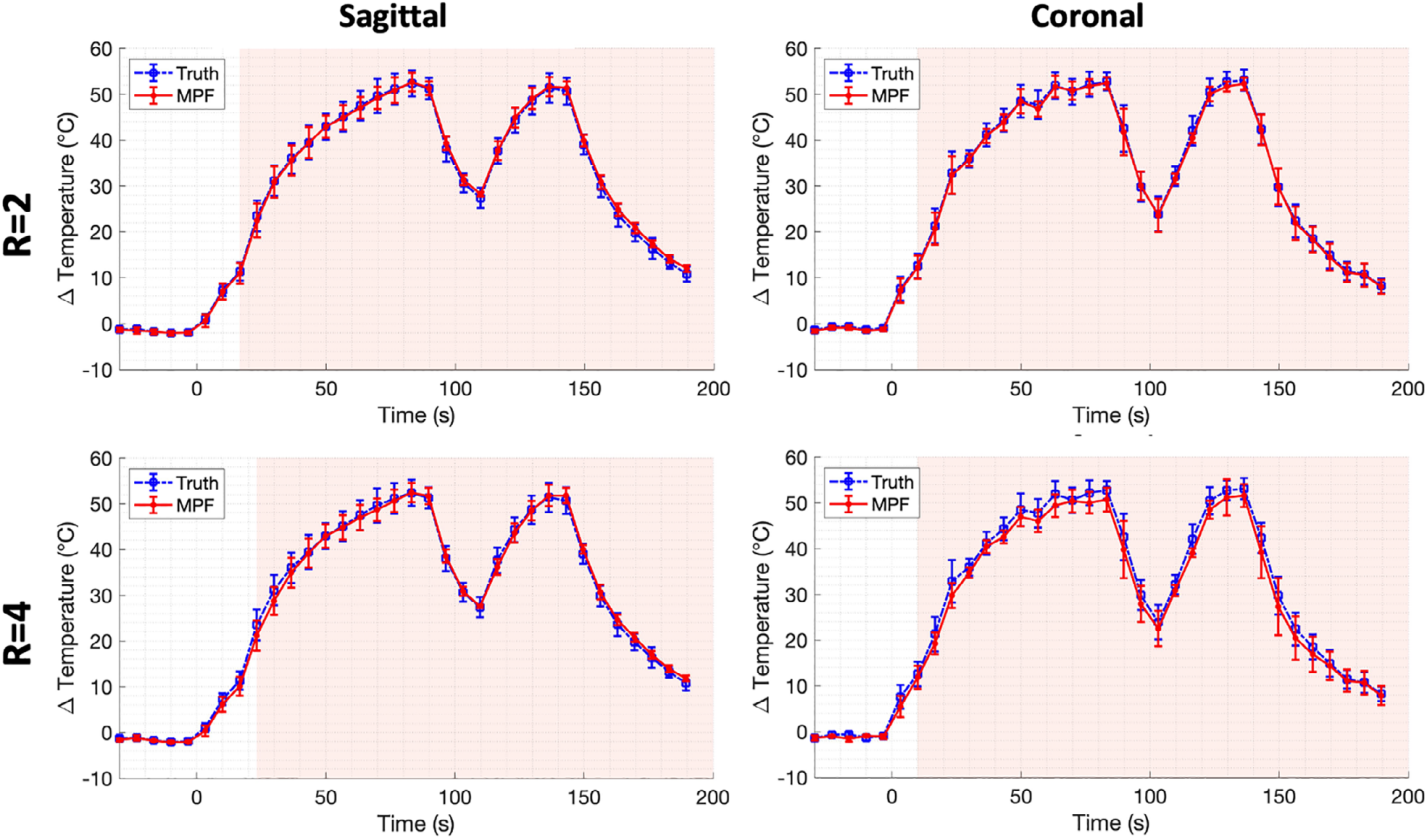
Temperature curves for the hottest voxel‐over‐time for in vivo data. *R* = 2 and *R* = 4 curves in both the sagittal and coronal views show good agreement, with slightly increased error in the coronal view at *R* = 4.

The MPF average temperature over time (± SD) for a 3 × 3 voxel region centered about the hottest voxel is shown in comparison with the “truth” in Figure 7. A summary of metrics comparing the “truth” and MPF averaged 3 × 3 voxel regions over the range of subsampling factors (*R* = 2–5) is shown in Table 2. Of note, sagittal images showed very good agreement (RMSE $\leq 1.1^{{}^{\circ}}C$) even up to the highest subsampling factor (*R* = 5). The maximum error over time between averaged regions surpassed $1.0^{{}^{\circ}}C$ when subsampling was *R* > 3. The coronal images showed increased error, especially for higher subsampling factors, as noted in Table 2 and visually evidenced in Figure 7. For example, for *R* = 4 and *R* = 5, RMSE values were $1.6^{{}^{\circ}}C$ and $2.3^{{}^{\circ}}C$ respectively, and maximum error surpassed $2.0^{{}^{\circ}}C$ when subsampling was *R* > 3. However, for more conservative subsampling (*R* = 2 and 3), RMSE values were less than $1.0^{{}^{\circ}}C$ for both sagittal and coronal views. Thus, when subsampling for *R* ≤ 3, the MPF approaches the aforementioned measurement accuracy (0.6°C and 0.8°C for coronal sagittal, respectively). For both views and all subsampling factors, no significant difference (*p* > 0.05) was found between the MPF and “truth” averaged regions over time using a two‐sample *t*‐test.

**TABLE 2:** Summary of in vivo results.

Subsampling factor	Sagittal		Coronal	
	RMSE ( 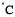 )	Max ΔT ( 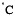 )	RMSE ( 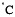 )	Max ΔT ( 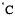 )
R=2	0.6	1.0	0.5	1.6
R=3	0.6	1.0	0.7	1.7
R=4	0.8	2.4	1.6	3.1
R=5	1.1	3.6	2.3	6.8

*Note*: All values were found by comparing the mean of a 3 × 3 ROI centered at the hottest voxel of the “true” temperature map with the mean of a 3 × 3 ROI centered at the hottest voxel of the MPF temperature map for each time point. ΔT = Truth−MPF.

**FIGURE 8:**
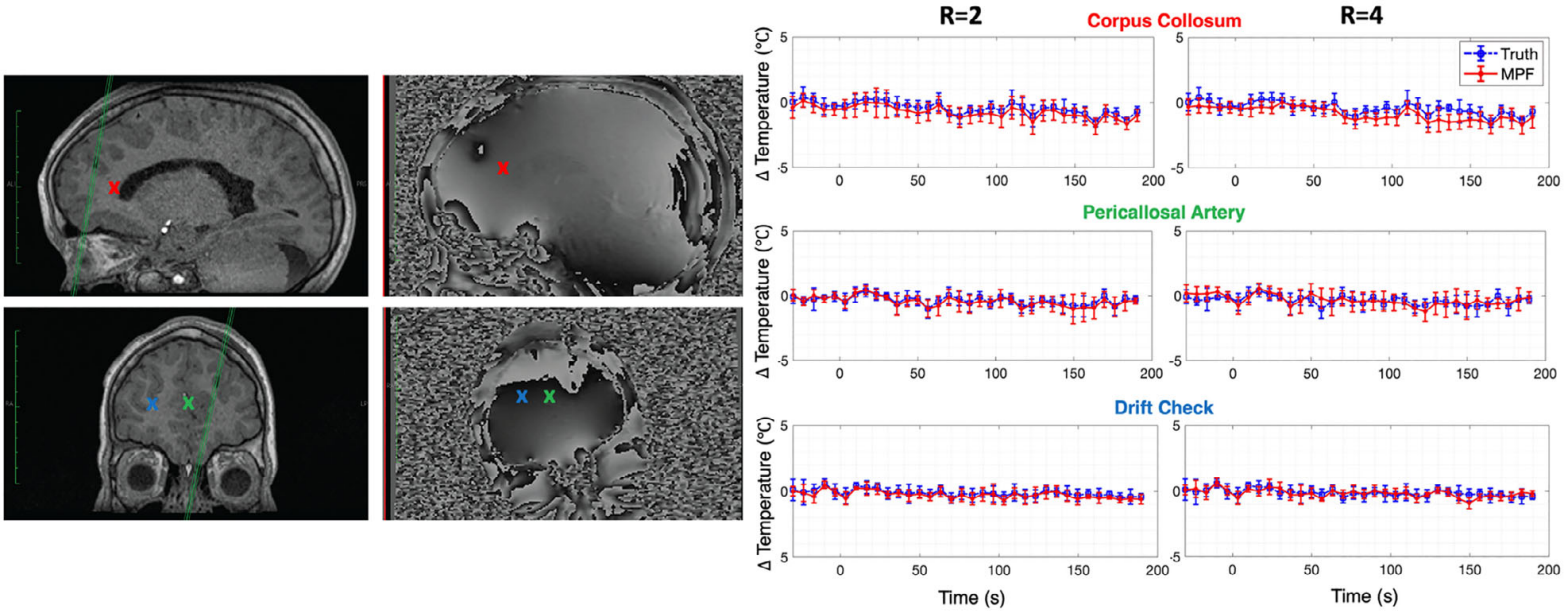
Temperature curves for the two safety points (corpus collosum, sagittal; pericallosal artery, coronal) and the drift check point (coronal) at both *R* = 2 and *R* = 4. Color coded crosses show points in the anatomical T_1_ (left) and phase (right) images. Note that locations in the anatomical T_1_ images are only approximate due to slightly altered slice orientation.

Structural T_1_‐weighted (Fast Spoiled Gradient‐Echo, FSPGR, acquired at 0.94 × 0.94 × 1.13 mm resolution, TR = 5.2 ms, TE = 1.5 ms, readout BW = 488 Hz/px, flip angle = 12°) and phase images are presented in Figure 8 showing the locations of the two safety points and the drift monitoring point. At each point, the MPF average temperature over time (± SD) for a 3 × 3 voxel region was again compared to the same region in the “truth” data (see Figure 8). These data are presented above the corresponding structural and phase images. Both the “truth” and the MPF data showed no temperature elevation in the selected safety points. The RMSE values at the sagittal safety point were 0.4°C (*R* = 2) and 0.8°C (*R* = 4). The RMSE at the coronal safety point was 0.3°C for *R* = 2 and 0.5°C *R* = 4. These values show good agreement with the aforementioned measurement accuracy of the MRTI data, suggesting heating was minimal and these values simply reflect measurement error. Little to no temperature drift was seen for both the “truth” and MPF data.

## 4 Discussion

A strategy is presented for achieving increased temporal resolution and/or spatial coverage during LITT heating of a brain phantom and in vivo brain tissue. In the phantom experiments, the same temporal resolution as the 12‐slice acquisition was achieved with good agreement, introducing the possibility of monitoring heating and organs at risk with larger FOV coverage during treatments. Alternatively, the larger FOV achieved using the MPF strategy could be traded for faster imaging, or a combination of both.

Using in vivo data from a clinical LITT case, two orthogonal slices of MRTI data were reconstructed using the MPF algorithm and a range of subsampling factors (*R* = 2–5), in this case using a pseudo‐random Gaussian subsampling strategy. Resulting temperature maps again showed excellent agreement in the 240 CEM_43_ area and hottest voxel regions showed low RMSE up to *R* = 3. In the coronal case, the RMSE remained low up to *R* = 5. The safety point and drift check indicated that the algorithm could provide accurate safety feedback for physicians and an estimate of the temperature drift during treatment. While there remains a need to show the efficacy of the MPF approach with in vivo volumetric measurements, as was demonstrated in the phantom experiments, these results suggest that the MPF strategy is sufficiently accurate to provide thermal dose maps with high levels of agreement for in vivo data with reduction factors in the range of *R* = 2–5. This can potentially enable significantly reduced acquisition times or acquisition of more slices in the same scan time.

The coronal in vivo data showed an increase in temperature error and variability compared to the “truth”. A comparison of the magnitude images with the temperature maps revealed that this error pattern approximately followed the path of the laser probe. It cannot be ruled out that some amount of water evaporation or air may have been created or induced through boiling given the high treatment temperatures [38, 39]. Additional in vivo studies could be helpful in determining the frequency of similar phenomena. Importantly, the elevated error at *R* = 4 for the coronal view did not noticeably affect the thermal dose calculation and regional comparison.

The pseudo‐random gaussian retrospective subsampling strategy utilized in the in vivo data may have contributed to an increase in MPF accuracy, as it sampled the center of *k*‐space for each time point. In contrast, the kz‐inside‐ky sampling order used for the phantom data results in even subsampling across *k*‐space for each time point. As shown previously, variable density subsampling for EPI approaches is possible but more challenging to employ [40] and such sampling may not be feasible for real‐time implementations of MPF. Further exploration of such strategies may allow for higher subsampling in the volumetric case.

A logical extension of this work is the implementation of the MPF strategy with *volumetric data* in vivo, which could produce a temporal resolution increase (i.e., faster imaging) for larger FOV. This endpoint is most similar to the phantom data presented here, where high temporal resolution was achieved at increasing volume FOV. If larger FOVs are not deemed necessary, faster acquisitions could instead be achieved by, for example, subsampling a 12‐slice acquisition with *R* = 2 or 3, achieving an acquisition time of 1.5–2.3 s for a thin 3D slab.

Future work should investigate the effect of different subsampling parameters, including higher reduction factors or varied subsampling patterns most suited to in vivo temperature imaging [22, 40]. Further developments to the Green's method, such as heterogenous tissue models and tissue‐specific thermal parameter optimization, can also be investigated [23]. More exhaustive modeling strategies, including the inclusion of perfusion or vessel models [25] or an analysis of the impact of modeling resolution [41] could possibly also improve accuracy. As noted in the methods, we found the most accurate results when excluding catheter cooling from our forward model. Additional phantom studies could be performed to identify what MRTI spatial resolution and temperature‐to‐noise ratio is needed to effectively characterize and model the effects of catheter cooling. Nevertheless, it appears that the implementation of Q in the basic forward model was sufficient to achieve acceptable accuracy compared to the fully‐sampled temperature maps. Additionally, we note that other strategies (such as the linear approximation method, described in, for example, Freeman et al. [21], or theoretical models for LITT applicator heating [42, 43]) exist to derive the Q map used in the forward temperature model in MPF. Future work will explore the interplay between Q derivation and image acquisition choices, like MRTI spatial and temporal resolution and undersampling methods [44], beyond this initial study.

Additional improvements to temperature imaging could also be made, such as the implementation of referenceless imaging to the acquired data [45]. Due to the low computational burden of the MPF approach (reconstruction times of 1 s per 3D time point and < 4 ms per two 2D slices), this strategy could be readily implemented into a real‐time pre‐clinical or clinical workflow to support higher spatial or temporal resolution temperature imaging after parameter and code optimization.

## 5 Conclusion

Reconstruction using the MPF algorithm was performed for both phantom and in vivo patient data, with acceptable DSC values in the volumetric phantom data (DSC ≥ 0.7) up to *R* = 3 and high DSC values in the 2D in vivo data (DSC > 0.95) for up to *R* = 5. Hottest voxel (or region of voxels)‐over‐time measurements showed good agreement between MPF and “truth” for a range of subsampling values. In vivo experiments showed good agreement at safety and drift check points. With additional developments in both imaging and modeling strategies, this strategy could permit increased FOV coverage or temporal resolution during clinical LITT treatments.

## Funding

This work was supported by National Institutes of Health, R01EB028316, R21EB033117, R21EB033638, S10OD018482; University of Utah.

## Supporting information


**Figure S1:** The first set of phantom “truth” and MPF reconstructed temperature maps (3D) at the hottest time point with overlayed 240 CEM_43_ contours. Rightmost images show the difference maps, where Difference = Truth−MPF. Three orthogonal views are shown for each trial (center: XY, right: XZ, bottom: YZ). Scale bar is 50 mm.
**Figure S2:** The third set of phantom “truth” and MPF reconstructed temperature maps (3D) at the hottest time point with overlayed 240 CEM_43_ contours. Rightmost images show the difference maps, where Difference = Truth−MPF. Three orthogonal views are shown for each trial (center: XY, right: XZ, bottom: YZ). Scale bar is 50 mm.
**Figure S3:** Visualization of the in vivo *k*‐space magnitude at a single time point with pseudo‐gaussian undersampling at both *R* = 2 and *R* = 5 in the phase encoding dimension. Note: filtering in the readout dimension was performed during vendor image processing of raw data and was already present in the DICOM data obtained for this work.

## Data Availability

The data that support the findings of this study are available from the corresponding author upon reasonable request.
