# Supplementary material for: Model Predictive Filtering MR Temperature Imaging for Laser‐Induced Interstitial Thermotherapy
Source: Magn Reson Med. 2026 Feb 15;95(6):3561–73. doi: 10.1002/mrm.70302 (PMC13049278; doi:10.1002/mrm.70302)
Supplement: Supplementary file 1 — Figure S1: The first set of phantom “truth” and MPF reconstructed temperature maps (3D) at the hottest time point with overlayed 240 CEM43 contours. Rightmost images show the difference maps, where Difference = Truth−MPF. Three orthogonal views are shown for each trial (center: XY, right: XZ, bottom: YZ). Scale bar is 50 mm. Figure S2: The third set of phantom “truth” and MPF reconstructed temperature maps (3D) at the hottest time point with overlayed 240 CEM43 contours. Rightmost images show the difference maps, where Difference = Truth−MPF. Three orthogonal views are shown for each trial (center: XY, right: XZ, bottom: YZ). Scale bar is 50 mm. Figure S3: Visualization of the in vivo k‐space magnitude at a single time point with pseudo‐gaussian undersampling at both R = 2 and R = 5 in the phase encoding dimension. Note: filtering in the readout dimension was performed during vendor image processing of raw data and was already present in the DICOM data obtained for this work. [file MRM-95-3561-s001.docx]

**Supporting Figures for:**

**Model Predictive Filtering MR Temperature Imaging for Laser-Induced Interstitial Thermotherapy**

**Joshua Marchant^1^, Robert J Bollo^2^, Dennis L Parker^1^, and Henrik Odéen^1^**

^1^Department of Radiology and Imaging Sciences, University of Utah, Salt Lake City, Utah, USA

^2^Department of Neurosurgery, University of Utah, Salt Lake City, Utah, USA


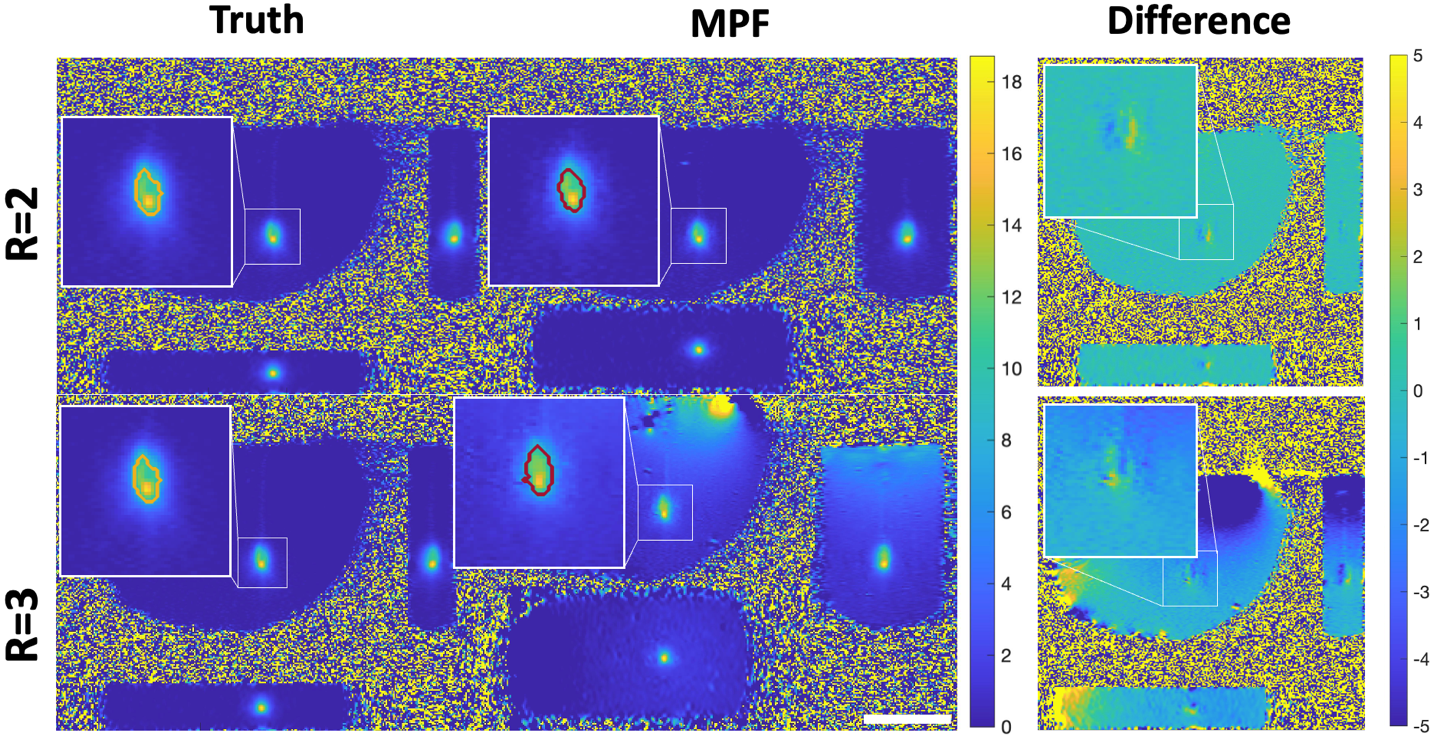


**Figure S1**: The first set of phantom “truth” and MPF reconstructed temperature maps (3D) at the hottest time point with overlayed 240 CEM_43_ contours. Rightmost images show the difference maps, where Difference = Truth – MPF. Three orthogonal views are shown for each trial (center: XY, right: XZ, bottom: YZ). Scale bar is 50 mm.


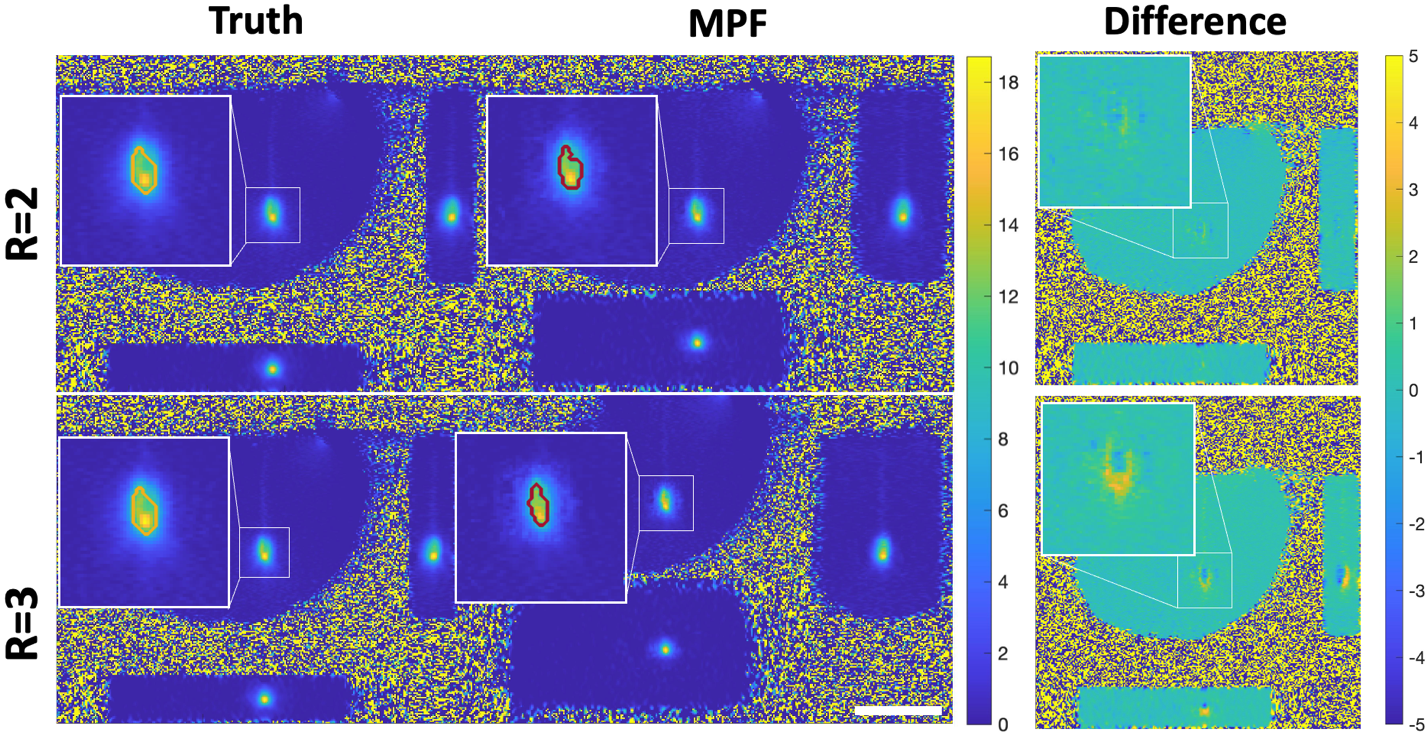


**Figure S2**: The third set of phantom “truth” and MPF reconstructed temperature maps (3D) at the hottest time point with overlayed 240 CEM_43_ contours. Rightmost images show the difference maps, where Difference = Truth – MPF. Three orthogonal views are shown for each trial (center: XY, right: XZ, bottom: YZ). Scale bar is 50 mm.


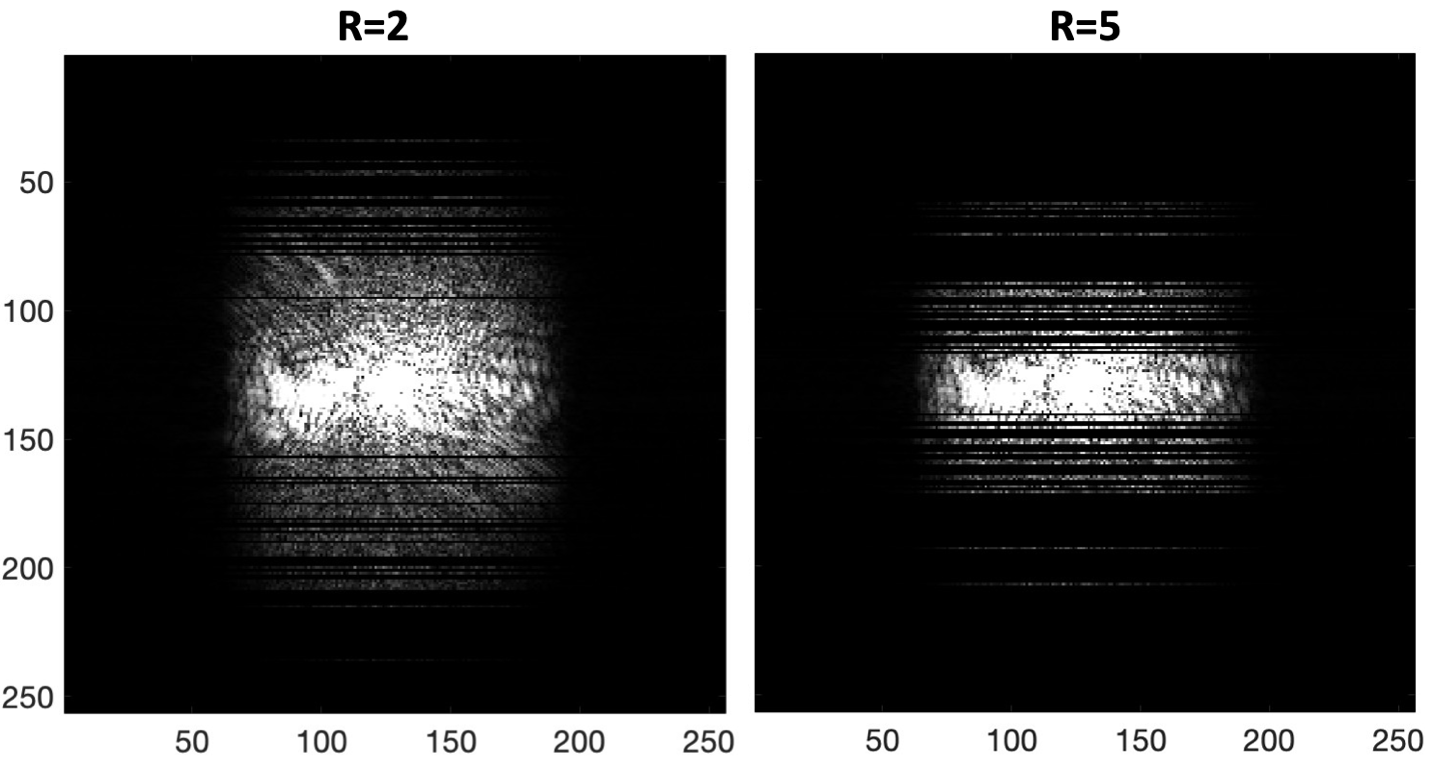


**Figure S3**: Visualization of the in vivo k-space magnitude at a single time point with pseudo-gaussian undersampling at both R=2 and R=5 in the phase encoding dimension. Note: filtering in the readout dimension was performed during vendor image processing of raw data and was already present in the DICOM data obtained for this work.
